# Down-regulation of sirtuin 3 is associated with poor prognosis in hepatocellular carcinoma after resection

**DOI:** 10.1186/1471-2407-14-297

**Published:** 2014-04-28

**Authors:** Jia-Xing Wang, Yong Yi, Yi-Wei Li, Xiao-Yan Cai, Hong-Wei He, Xiao-Chun Ni, Jian Zhou, Yun-Feng Cheng, Jian-Jun Jin, Jia Fan, Shuang-Jian Qiu

**Affiliations:** 1 Liver Cancer Institute, Zhongshan Hospital, Fudan University, Key Laboratory for Carcinogenesis & Cancer Invasion, The Chinese Ministry of Education, Shanghai, People’s Republic of China; 2 Biomedical Research Center, Zhongshan Hospital, Fudan University, Shanghai, People’s Republic of China

**Keywords:** Sirtuin, Hepatocellular carcinoma, Prognosis

## Abstract

**Background:**

Sirtuin 3 (Sirt3), one of the seven Sirtuins family members, plays critical roles in the progression of multiple cancer types. However, its role in the prognosis of hepatocellular carcinoma (HCC) has not yet been investigated systematically.

**Methods:**

The correlation of Sirtuins expression with prognosis of HCC was determined by immunohistochemistry (IHC) in a large HCC patient cohort (n = 342). Expression of Sirt3 in tumoral and peritumoral tissues of HCC patients were further determined by western blotting (WB).

**Results:**

IHC and WB studies both showed a decreased expression of Sirt3 in tumoral tissues compared with peritumoral tissues (*P* = 0.003 for IHC, *P* = 0.0042 for WB). Decreased expression of Sirt3 in both tumoral and peritumoral tissues was associated with increased recurrence probability and decreased overall survival rate by univariate analyses (intratumoral Sirt3: *P* = 0.011 for TTR, *P* = 0.001 for OS; peritumoral Sirt3: *P* = 0.017 for TTR, *P* = 0.023 for OS), the prognostic value was strengthened by multivariate analyses (intratumoral Sirt3: *P* = 0.031 for TTR, *P* = 0.001 for OS; peritumoral Sirt3: *P* = 0.047 for TTR, *P* = 0.031 for OS). Intratumoral Sirt3 also showed a favorable prognostic value in patients with BCLC stage A (TTR, *P* = 0.011; OS, *P* < 0.001). In addition, we found that IHC studies of other sirtuin members showed a decreased expression of Sirt2, Sirt4 and Sirt5 and an increased expression of Sirt1, Sirt6 and Sirt7 in intratumoral tissues compared with peritumoral tissues. In contrast to Sirt3, other members did not showed a remarkable correlation with HCC prognosis.

**Conclusions:**

Down-regulation of intratumoral and peritumoral Sirt3 were both associated with poor outcome in HCC, moreover, intratumoral Sirt3 was a favorable prognostic predictor in early stage patients.

## Background

Hepatocellular carcinoma (HCC), with rising incidence in the west, is the third leading cause of cancer-related death worldwide [1]. Although many advances in HCC therapy had been reached, surgical resection and liver transplantation remain the most reliable curative treatment modalities for selected patients. One of the major obstacles for improved outcome after resection is the high frequency of recurrence. It was proposed that reactive oxygen species (ROS) produced by mitochondria was participated in HCC progression and metastasis, through promoting DNA damage or altering cellular signaling pathways [2-5]. Recently, Sirt3 has emerged as a critical modulator of mitochondria function by reducing mitochondria membrane potential and ROS levels [6-9].

The Sirtuins, a family of orthologues of yeast silent information regulator 2 (Sir2) found in a wide range of organisms from bacteria to human, regulate metabolism; cellular proliferation and survival; stress resistance and apoptosis, and participate in metabolic; cardiovascular and neurodegenerative diseases; inflammatory and cancers [10-12]. Sirt3, a member of the family, functions mainly as the primary mitochondrial deacetylase that modulates mitochondrial metabolic and oxidative stress regulatory pathways [9,13,14]. Published data revealed that Sirt3 was implicated in tumor progress [15,16], mainly through mediating the suppression of hypoxia inducible factor 1α (HIF-1α) and inhibiting mitochondrial ROS production [17,18]. The proliferation-suppressor role of Sirt3 was confirmed in multiple cancer types, including breast cancer and colon cancer, both *in vitro* and *in vivo*[6,18]; it was also reported that Sirt3 could inhibit HCC cell growth through reducing Mdm2-mediated p53 degradation [19]. However, the expression status of Sirt3 in human HCC specimens is still ambiguous and the relationship between Sirt3 expression and cancer prognosis is still unclear. Hence, further intensive investigation is substantial.

In this study, we evaluated the expression status of the Sirtuin family members (Sirt1-7), mainly focusing on mitochondria member Sirt3, in a large HCC cohort by IHC staining. Then we further investigated the expression of the Sirt3 in HCC specimens.

## Methods

### Patients and TMA construction

A total of 342 HCC patients were enrolled in this study at the Liver Cancer Institute of Fudan University (Shanghai, China) between 2007 and 2008, and informed consent was obtained from each patient. The including criteria and postoperative follow-up procedure were described previously [20,21]. The clinicopathological characteristics of the patients were summarized in Table 1. The Barcelona Clinic Liver Cancer (BCLC) staging system was applied to classify the disease stage [22]. Tumor differentiation was graded by the Edmond-son-Steiner grading system [23]. Time to recurrence (TTR) and overall survival (OS) time were defined as the interval from primary surgical resection to the first recurrence or death, respectively. The study was approved by the Zhongshan Hospital Research Ethics Committee (Fudan University, China).

**Table 1:** Clinicopathologic features of the patients

Age, y, median (range)	53 (10 ~ 70)
Gender (male/female)	290/52
Hepatitis infection (no/yes)	94/248
Liver cirrhosis (no/yes)	38/304
AFP, ng/ml, median (range)	98.5 (0 ~ 60500)
γ-GT, U/L, median (range)	57 (7 ~ 693)
ALT, U/L, median (range)	39 (8 ~ 949)
Child-Pugh score (A/B)	342/0
Tumor size, cm, median (range)	4 (1.0 ~ 21.0)
Tumor number (single/multiple)	298/44
Tumor capsule (yes/no)	183/159
Tumor differentiation (I/II/III/IV)	7/248/85/2
Tumor thrombi (no/yes)	249/93
TNM stage (I/II&III)	154/188
BCLC stage (A/B&C)	233/109
Prophylactic therapy (none/TACE/immunotherapy^a^)	342/0/0
Post-recurrence therapy (none/TACE/regional^b^/resection/others^c^)	221/121/0/0

***Abbreviations*:** AFP, alpha-fetoprotein; γ-GT, gamma-glutamyl transpeptidase; ALT, alanine transaminase; TNM, tumor-node-metastasis; BCLC, Barcelona Clinic Liver Cancer; TACE, transcatheter arterial chemoembolization.

a. immunotherapy: interferon-α or thymosin therapy.

b. regional: radio frequency ablation (RFA), percutaneous ethanol injection therapy (PEI) or microwave ablation (MA); others: traditional Chinese medicine etc.

c. others: thrombi from hepatic vein, inferior caval vein or intrahepatic ducts.

### Tissue immunohistochemistry and evaluation system

Tissue microarray (TMA) was constructed as described previously [24]. IHC was carried out according to appropriate protocols as described in our previous reports [21]. Briefly, slides were deparaffinized in xylene and hydrated through a graded alcohol series before being placed in blocking solution to inhibit endogenous peroxidase activity. The slides were incubated with primary antibody (Sirt1 1:100, Epitomics; Sirt2 1:200, Epitomics; Sirt3, 1:200 Abgent; Sirt4, 1:200 Abgent; Sirt5, 1:100 Abgent; Sirt6, 1:150 Abgent; Sirt7, 1:100 Abgent) at 4°C overnight. Slides were then applied in the detection system of Elivision™ Plus Kit and DAB, followed by counterstaining with hematoxylin.

As a mitochondrial factor, we evaluated cytoplasmic expression of Sirt3 in HCC. A scoring system was applied as previously described with some modifications [25-27]. In brief, a staining index for each case was determined by multiplying the score for intensity of cytoplasmic staining (none = 0, weak = 1, strong = 2) with the score for proportion of tumor cytoplasma stained (<5% = 0, 5%-25% = 1, 25%-50% = 2, 50%-75% = 3, >75% = 4) [21,26]. The results were confirmed by two experienced pathologists who were blinded to the clinicopathologic data of the patients.

### Western blot analysis

The immunoblotting was carried out as previously described [28]. In brief, about thirty micrograms of proteins extracted from paired HCC and its adjacent tissues were separated by SDS-PAGE, after which the protein was transferred to polyvinylidene fluoride membranes (Millipore), membrane-bound Sirt3 was detected with rabbit anti-human Sirt3 (1:1000, Epitomics). GAPDH (1:5000, KANGCHENG) was used as an internal control. WB analysis was proceeded by the relative expression of Sirt3 in peritumoral tissues compared with tumoral tissues in each case of 51 specimen, by using GAPDH as an internal control. Furthermore, we detected the protein level of superoxide dismutase 2 (SOD2) (1:1000, Epitomics) and Sirt3 via WB analysis in another independent 15 HCC specimen.

### Statistics

Statistical analyses were carried out using Statistical Package of the Social Sciences (SPSS version 17.0). χ2 test and paired *t* test were done as appropriate. Univariate analyses were done using the Kaplan-Meier method and compared by the long-rank test. Cox multivariate analysis was used to adjust for potentially confounding variables and to determine the independent prognostic factors. The “minimum *P* value” approach [24,29] was used to get optimal cut-off for the best separation between groups of patients related to TTR or OS. Unless otherwise specified, all data were analyzed using two-tail test and *P* < 0.05 was considered statistically significant.

## Results

### Patient profile

The detailed clinicopathological characteristics of the patients are supplied in Table 1. The median follow-up was 42.9 months (range, 0.43-61.83 months; SD, 18.8 months). At the last follow-up (March 31st, 2012), 158 patients (46.2%) had recurrence. 125 patients (36.5%) died of recurrence (n = 103) or cirrhosis related complications without recurrence (n = 22) (These data of follow up of patients were not shown in Table 1).

### Immunohistochemical expression pattern of Sirt3 in paired tumoral and peritumoral tissues

**Figure 1:**
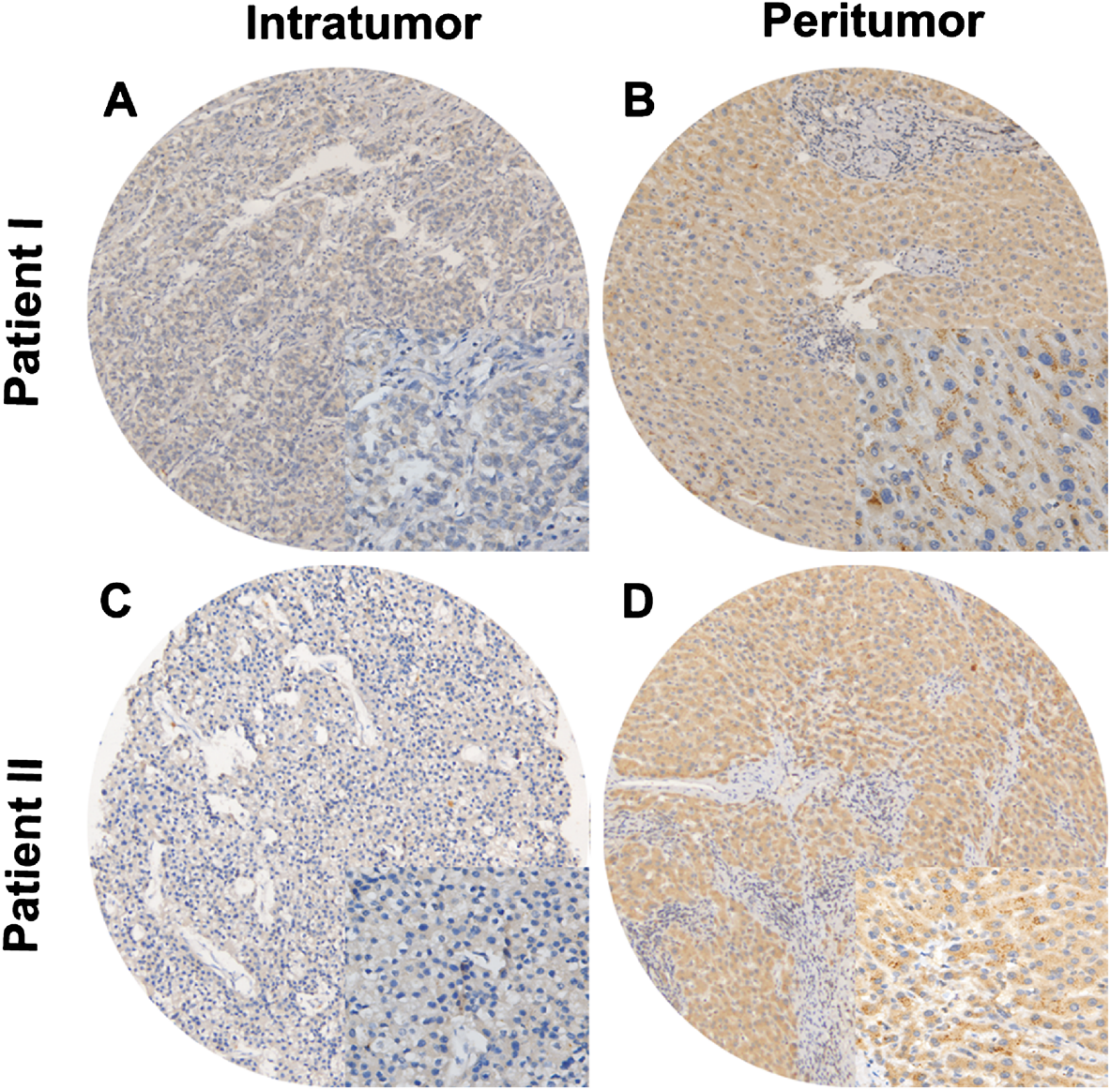
**Representative immunohistochemical staining of Sirt3.** The micrographs showed weak staining of Sirt3 in Patient I **(A)**, nearly negative staining in Patient II **(C)** in tumor tissues, and strong staining of peritumoral liver tissues **(B, D)** in patient I and II. (magnification100× & 400×).

We found that the majorities of tumoral and peritumoral tissues showed diffuse cytoplasmic expression pattern of Sirt3 (Figure 1). Compared with paired peritumoral tissues, tumoral tissues had significantly down-regulated expression of Sirt3 (mean, 4.07 vs. 4.27, *P* = 0.003). Representative cases of Sirt3 IHC staining were show in Figure 1. The expression pattern of other sirtuin members was described in the Supplementary Information (See Additional file 1: Figure S1).

### Prognostic significance of Sirt3 for HCC

By using the “minimum *P* value” approach, scoring value of 2 and 4 are the best cut-off value for intratumoral and peritumoral Sirt3, respectively (See Additional file 2: Table S1).

**Figure 2:**
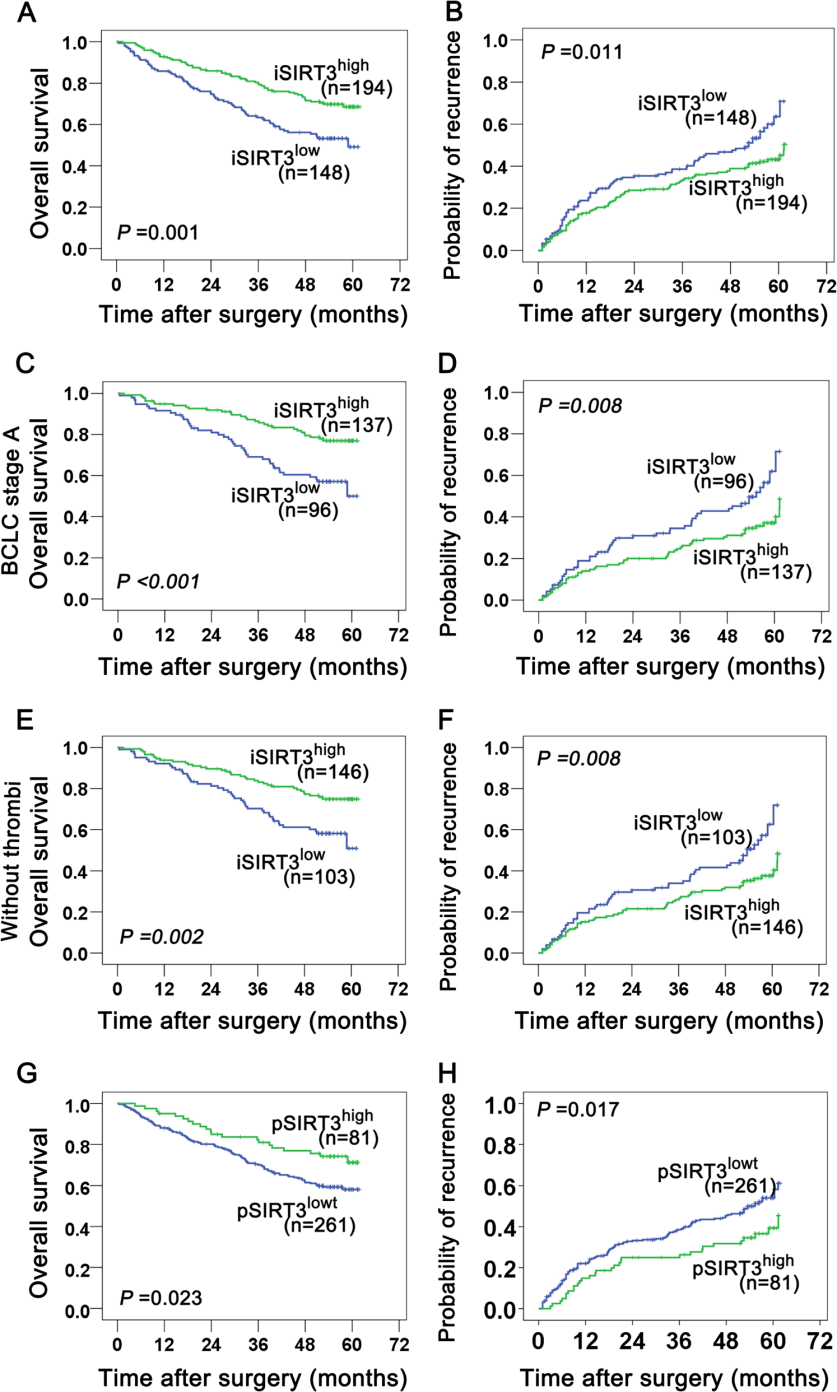
**Kaplan-Meier analysis of OS and TTR for the expression levels of Sirt3.** Kaplan-Meier analysis of OS and TTR for the expression levels of intratumoral Sirt3 **(A and B)** and peritumoral Sirt3 **(G and H)**. Subgroup analysis of Sirt3 expression in relation to OS and TTR indicated that intratumoral Sirt3 had prognostic role when classified by BCLC stage A **(C and D)** and without thrombi **(E and F)**.

On univariate analysis, patients with lower expression of Sirt3 in tumor were prone to lower OS (Figure 2A, *P* = 0.001) and shorter TTR (Figure 2B, *P* = 0.011). Other clinicopathologic factors associated with OS or TTR were shown in Table 2. Factors that showed significance by univariate analysis were enrolled as covariate in a multivariate Cox proportional hazards model. Multivariate analysis revealed that intratumoral Sirt3 was an independent prognostic indicator for OS (Table 2, *P* = 0.001), and retained the prognostic power for predicting recurrence (Table 2, *P* = 0.031). Furthermore, we found that intratumoral Sirt3 showed prognostic role in BCLC stage A patients (Figure 2C and D, OS, *P* < 0.001; TTR, *P* = 0.008), and in no vascular invasion subgroups (Figure 2E and F, OS, *P* = 0.002; TTR, *P* = 0.008). Intratumoral Sirt3 also showed prognostic role in other groups when classified by the following variables (Table 3): large tumor (>5 cm) (OS, *P* = 0.003; TTR, *P* = 0.046), single tumor (OS, *P* < 0.001; TTR, *P* = 0.011), tumor with encapsulation (OS, *P* = 0.002; TTR, *P* = 0.036), tumor differentiation grade I-II (OS, *P <* 0.001; TTR, *P* = 0.007).

**Table 2:** Univariate and multivariate analyses of Sirt3 associated with recurrence and survival

	**TTR**			**OS**		
	**Univariate**	**Multivariate**		**Univarate**	**Multivariate**	
	***P*** **value**	**H.R. (95% CI)**	***P*** **value**	***P*** **value**	**H.R. (95% CI)**	***P*** **value**
BCLC stage A						
γ-GT, U/L (≤54 vs >54)	0.003	1.646 (1.091–2.484)	0.016	0.003	1.718 (1.051–2.810)	0.028
AFP, ng/ml (≤20 vs >20)	0.013	1.574 (1.033–2.398)	0.032	0.060	NA	NA
Tumor size, cm (≤5 vs >5)	<0.001	2.292 (1.515–3.470)	<0.001	<0.001	3.150 (1.952–5.084)	<0.001
Tumor number (single vs multiple)	0.948	NA	NA	0.726	NA	NA
Tumor capsule (yes vs no)	0.296	NA	NA	0.690	NA	NA
Differentiation (well vs poor)	0.429	NA	NA	0.550	NA	NA
Mean density score (low vs high)						
iSirt3(2)	0.008	0.591 (0.396–0.884)	0.011	<0.001	0.404 (0.251–0.650)	<0.001
pSirt3(4)	0.020	0.538 (0.316–0.914)	0.022	0.278	NA	NA
TMA assays						
Age, year (≤52 vs >52)	0.173	NA	NA	0.787	NA	NA
Gender (female vs male)	0.616	NA	NA	0.654	NA	NA
HBV infection (no vs yes)	0.903	NA	NA	0.168	NA	NA
Liver cirrhosis (no vs yes)	0.803	NA	NA	0.225	NA	NA
ALT, U/L (≤75 vs >75)	0.177	NA	NA	0.225	NA	NA
γ-GT, U/L (≤54 vs >54)	<0.001	1.563 (1.121–2.177)	0.008	<0.001	1.471 (1.003–2.159)	0.046
AFP, ng/ml (≤20 vs >20)	0.004	1.474 (1.048–2.072)	0.023	0.014	1.379 (0.936–2.031)	0.098
Tumor size, cm (≤5 vs >5)	<0.001	1.830 (1.314–2.549)	<0.001	0.000	2.919 (2.019–4.220)	<0.001
Tumor number (single vs multiple)	0.057	NA	NA	0.069	NA	NA
Tumor capsule (yes vs no)	0.096	NA	NA	0.167	NA	NA
Differentiation (well vs poor)	0.162	NA	NA	0.124	NA	NA
Tumor thrombi (no vs yes)	0.001	1.506 (1.072–2.116)	0.021	<0.001	1.658 (1.146–2.399)	0.009
TNM stage (I vs II/III)	<0.001	NA	NA	<0.001	NA	NA
BCLC stage (A vs B/C)	0.001	NA	NA	<0.001	NA	NA
Mean density score (low vs high)						
iSirt3(2)	0.011	0.705 (0.514–0.967)	0.031	0.001	0.558 (0.392–0.795)	0.001
pSirt3(4)	0.017	0.674 (0.450–1.009)	0.047	0.023	0.611 (0.381–0.979)	0.031

Note: Univariate analysis was calculated by the Kaplan–Meier method (the log-rank test). Multivariate analysis was done using the Cox multivariate proportional hazards regression model in a stepwise manner (backward, likelihood ratio). AFP, a-fetoprotein; 95% CI, 95% confidence interval; γ-GT, γ-glutamyl transferase; HR, hazard ratio; NA, not applicable; OS, overall survival; TTR, time to recurrence;TNM, tumor-node-metastasis; BCLC, Barcelona Clinic Liver Cancer.

**Table 3:** Survival analysis of Sirt3 expression in HCC patients when stratified by clinicopathologic factors

**Variables**		**iSirt3-TTR**	**iSirt3-OS**	**pSirt3-TTR**	**pSirt3-OS**
		***P*** **value**	***P*** **value**	***P*** **value**	***P*** **value**
TNM					
I	154	0.110	0.059	0.078	0.100
II/III	188	0.066	0.004	0.020	0.031
BCLC					
A	233	0.008	< 0.001	0.020	0.278
B/C	109	0.690	0.366	0.204	0.020
HBV infection					
No	94	0.037	0.127	0.343	0.217
Yes	248	0.095	0.003	0.033	0.051
Cirrhosis					
No	303	0.029	0.003	0.046	0.059
Yes	38	0.151	0.076	0.131	0.148
AFP					
≤20	132	0.075	0.078	0.371	0.334
>20	210	0.104	0.006	0.049	0.079
γ-GT (U/L)					
≤54	163	0.639	0.208	0.025	0.111
>54	179	0.012	0.002	0.384	0.164
Tumor size					
≤5	229	0.127	0.045	0.094	0.093
>5	113	0.046	0.003	0.062	0.108
Tumor number					
1	297	0.011	*<* 0.001	0.016	0.023
≥2	44	0.547	0.903	0.566	0.585
Tumor thrombi					
No	249	0.008	0.002	0.015	0.263
Yes	93	0.706	0.154	0.386	0.024
Tumor capsule					
Yes	183	0.036	0.002	0.200	0.191
No	159	0.209	0.124	0.035	0.064
Differentiation					
Well	255	0.007	*<* 0.001	0.009	0.028
Poor	87	0.856	0.879	0.838	0.535

Note: χ2 tests for all the analysis. AFP, alpha-fetoprotein, γ-GT, γ-glutamyl transferase. OS, overall survival; TTR, time to recurrence.

Meanwhile, patients with higher expression of Sirt3 in peritumoral tissues were prone to higher OS (Figure 2G, *P* = 0.023) and longer TTR (Figure 2H, *P* = 0.017), and multivariate analysis also revealed peritumoral Sirt3 had independent prognostic value for both OS (Table 2, *P* = 0.031) and TTR (Table 2, *P* = 0.047). Peritumoral Sirt3 also showed prognostic role in groups when classified by the following variables (Table 3): single tumor (OS, *P* = 0.023; TTR, *P* = 0.016), tumor differentiation grade I-II (OS, *P* = 0.028; TTR, *P* = 0.009). The aforementioned results suggested that down-regulation of intratumoral and peritumoral Sirt3 were both associated with unfavorable prognostic performance of HCC.

The prognostic value of other Sirtuin members were shown in the Supplementary Information (See Additional file 3: Table S2).

### Correlation between Sirt3 and clinicopathological features

Both tumoral and peritumoral Sirt3 expression level were not correlated with tumor size, tumor numbers, differentiation, encapsulation or vascular invasion. We found that Sirt3 expression level in peritumoral tissues was associated with AFP (Table 4). Peritumoral tissues with higher AFP were prone to have lower expression of Sirt3.

**Table 4:** Correlation of clinicopathologic characteristics with Sirt3 expression

		**iSirt3**			**pSirt3**		
**Characteristics**		**Low**	**High**	***P*** **value**	**Low**	**High**	***P*** **value**
Gender	Male	124	166	0.649	219	71	0.632
	Female	24	28		41	11	
Age (Years)	≤52	75	93	0.616	135	32	0.051
	>52	73	101		125	49	
HBV infection	No	47	47	0.122	68	26	0.296
	Yes	101	147		192	55	
Liver cirrhosis	No	132	172	0.877	231	72	0.991
	Yes	16	22		29	9	
ALT (U/L)	≤75	127	176	0.157	229	73	0.613
	>75	21	18		31	8	
AFP (ng/ml)	≤20	51	81	0.170	90	42	0.005
	>20	97	113		170	39	
γ-GT (U/L)	≤54	63	100	0.100	120	43	0.275
	>54	85	94		140	38	
Tumor size (cm)	≤5	95	134	0.342	174	55	0.870
	>5	53	60		86	26	
Tumor number	1	132	166	0.322	229	68	0.333
	≥2	16	28		31	13	
Tumor thrombi	No	103	146	0.244	188	61	0.595
	Yes	45	48		72	20	
Encapsulation	Yes	72	111	0.115	190	64	0.368
	No	76	83		70	17	
Differentiation	Well	109	146	0.735	136	47	0.285
	Poor	39	48		124	34	
TNM stage	I	64	90	0.562	122	32	0.242
	II/III	84	104		138	49	
BCLC stage	A	96	137	0.258	178	55	0.925
	B/C	52	57		82	26	

Note: χ2 tests for all the analysis. AFP, alpha-fetoprotein; γ-GT, γ-glutamyl transferase ; OS, overall survival ; TTR, time to recurrence.

### Sirt3 expression in HCC and peritumoral tissues

**Figure 3:**
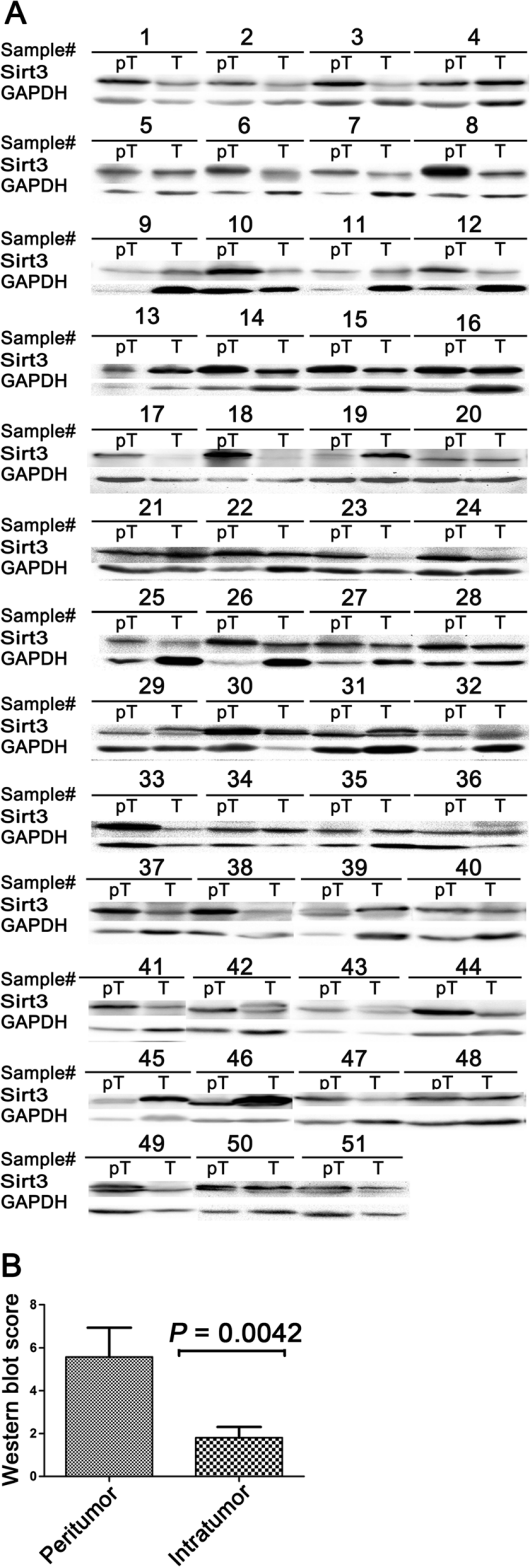
**Western blot analysis of Sirt3 in HCC and peritumoral liver tissues. (A)** WB detection of Sirt3 protein in 51 paired tumors (T) and peritumoral tissues (pT). GAPDH served as a loading control. **(B)** Sirt3 protein level obtained by densitometric scaning was significantly down-regulated in tumors compared to peritumoral tissues (*P* = 0.0042).

By WB detection of Sirt3 in 51 paired tumoral and peritumoral tissues, Sirt3 was significantly down-regulated in tumoral tissues compared to peritumoral tissues (Figure 3, *P* = 0.0042). As shown in Additional file 4, 9 of 51 specimen had higher expression of Sirt3 in tumor compared with paired peritumoral tissues, by using GAPDH as an internal control. In the 9 cases as mentioned above, 3 had recurrence, while 12 of 42 cases which showed high expression of Sirt3 in peritumoral tissues had recurrence (*P* = 0.776). Meanwhile, no difference was shown regarding the disease stage in 9 cases compared with residual 42 cases (See Additional file 5). This result was in accordance with the IHC analysis as mentioned and was supported by previous studies [19,30].

### Correlation between the expression of Sirt3 and SOD2 in HCC and peritumoral tissues

By WB detection of Sirt3 and SOD2 in independent 15 paired tumoral and peritumoral tissues, we showed that the expression of SOD2 was positively correlated with Sirt3 (r = 0.5418, *P* = 0.037) (See Additional file 6).

## Discussion

We systematically investigated the expression pattern and prognostic importance of the Sirtuins family for HCC undergoing radical resection for the first time. After a comprehensive analysis by IHC studies, we found that Sirt3 had more prognostic value.

In this study, we reported the down-regulation of Sirt3 in tumoral specimens compared with peritumoral tissues at the protein level, both by IHC staining and WB analysis (Figure 1 and Figure 3). The discrepancy of Sirt3 expression between tumoral and peritumoral tissues was consistent with the results of other cancer studies [6,19]. To our knowledge, ROS act as the second messengers to stimulate cell proliferation, apoptosis, and gene expression at the submicroscopic level, and excessively elevated levels of ROS can produce oxidative stress which leads to a variety of diseases, including cancer, aging, and neurologic disorders [31]. It has been proposed that Sirt3 regulated mitochondrial acetylation and ROS generation, and therefore mediated the tumor-inhibiting role in cancer. In consistent with this hypothesis, we found that the decreased expression level of intratumoral Sirt3 could independently predict elevated risks of tumor recurrence and patients’ death.

Of note, Sirt3 reduces cellular ROS levels via SOD2, a major mitochondrial antioxidant enzyme [32,33]. Sirt3 deacetylates two critical lysine residues on SOD2 and promotes its antioxidative activity [34]. Importantly, the abilities of SOD2 to reduce cellular ROS and promote oxidative stress resistance is greatly enhanced by Sirt3. In consistent with this relationship between SOD2 and Sirt3, our results showed that the expression of SOD2 was also correlated with that of Sirt3 in 15 HCC specimens, which supported that Sirt3 may reduce the expression level of ROS via the activation of SOD2.

In recent years, high recurrence rate remains a major barrier to improve postoperative outcome of HCC. So the early prediction of recurrence in HCC patients after resection is obviously important for timely treatment, which may lead to better clinically outcome, especially in patients with early stage HCC [35]. Compared to the advanced-stage HCC, the prognosis of early-stage HCC is far from homogenous, and lacks ideal indicators [36,37]. Now our results showed that in BCLC stage A or no vascular invasion group of patients, intratumoral Sirt3 expression showed the ability to predict the risk of recurrence and patient survival (Figure 2). Its independent prognostic value in early BCLC stage HCC patients is of clinical importance, which maybe a useful biomarker to identify patients who should be monitored frequently and then be subjected to adjuvant therapy like antioxidant therapy [38].

In our study, the patients with lower intratumoral Sirt3 expression showed not only higher recurrence but shorter survival times after curative resection, which was partly consistent with the results of a previous study [30]. The different results of recurrence in multivariate analysis between us may partly due to the different scoring systems of IHC evaluation and the methods of the definition of cutting off score. However, both results suggested intratumoral Sirt3 as an independent prognostic biomarker for OS in HCC patients after resection.

Recently, many studies revealed that robust production of ROS played important roles in hepatocarcinogenesis, which could directly induce DNA damage and alter gene expression [39,40]. Furthermore, there existed a high incidence of intrahepatic metastasis and recurrence after resection [41-43], which suggested that peritumoral environment was important. In our results, low expression of peritumoral Sirt3 was also associated with HCC recurrence and poor survival after hepatectomy. Therefore, peritumoral Sirt3 may serve as a protector of recurrence of HCC through preventing the generation of ROS. These results highlight the important role of remanent liver in recurrence and metastasis, and will be helpful in shaping postoperative strategy for the prevention of recurrence after hepatectomy.

## Conclusions

This present study indicated that both intratumoral and peritumoral Sirt3 expression were associated with prognosis in HCC. Moreover, we demonstrated that the down-regulation of Sirt3 in HCC indicated aggressive tumor behaviors and predicted an unfavorable clinical outcome. Also, Sirt3 may be a useful biomarker to identify the BCLC stage A group of patients at high risk of post-operative recurrence. Therefore, further study elucidating the molecular pathogenesis of the multi-facet Sirt3 in HCC may lead to more effective and specific therapies against this intractable cancer.

## Abbreviations

Sirt3: Sirtuin 3; HCC: Hepatocellular carcinoma; IHC: Immunohistochemistry; WB: Western blotting; ROS: Reactive oxygen species; HIF-1α: Hypoxia inducible factor 1α; BCLC: Barcelona clinic liver cancer; TTR: Time to recurrence; OS: Overall survival; TMA: Tissue microarray; SPSS: Statistical package of the social sciences; SOD2: Superoxide dismutase 2.

## Competing interests

The authors declare that they have no competing interests.

## Authors’ contributions

Conceived and designed the experiments: WJX, YY and QSJ. Performed the experiments: WJX. Analyzed the data: WJX, YY, LYW, CXY, HHW, NXC, ZJ, CYF, JJJ, FJ. Wrote the paper: WJX. All authors read and approved the final manuscript.

## Pre-publication history

The pre-publication history for this paper can be accessed here:

http://www.biomedcentral.com/1471-2407/14/297/prepub

## Supplementary Material

Additional file 1: Figure S1 Representative IHC staining of Sirt1, 2, 4, 5, 6, 7. The micrographs showed nearly negative cytoplasma staining of Sirt2, Sirt4, Sirt5 in tumor tissues, and Sirt1, Sirt6, Sirt7 of peritumoral liver tissues in HCC patients. (magnification100× & 400×). Click here for file

Additional file 2: Table S1 X-tile for minimum *P* value. Click here for file

Additional file 3: Table S2 Univariate and multivariate analyses of prognostic factors. Click here for file

Additional file 4 The ratio of pT/T of Sirt3 by WB in 51 HCC patients. Click here for file

Additional file 5 Correlation between the ratio of iSirt3/pSirt3 and clinicopathologic characteristics in 51 patients. Click here for file

Additional file 6 **Correlation between expression level of Sirt3 and SOD2 in HCC patients.** 15 cases were studied. Click here for file
