# Supplementary figures and images for: Down-regulation of sirtuin 3 is associated with poor prognosis in hepatocellular carcinoma after resection
Source: BMC Cancer. 2014 Apr 28;14:297. doi: 10.1186/1471-2407-14-297 (PMC4021365; doi:10.1186/1471-2407-14-297)

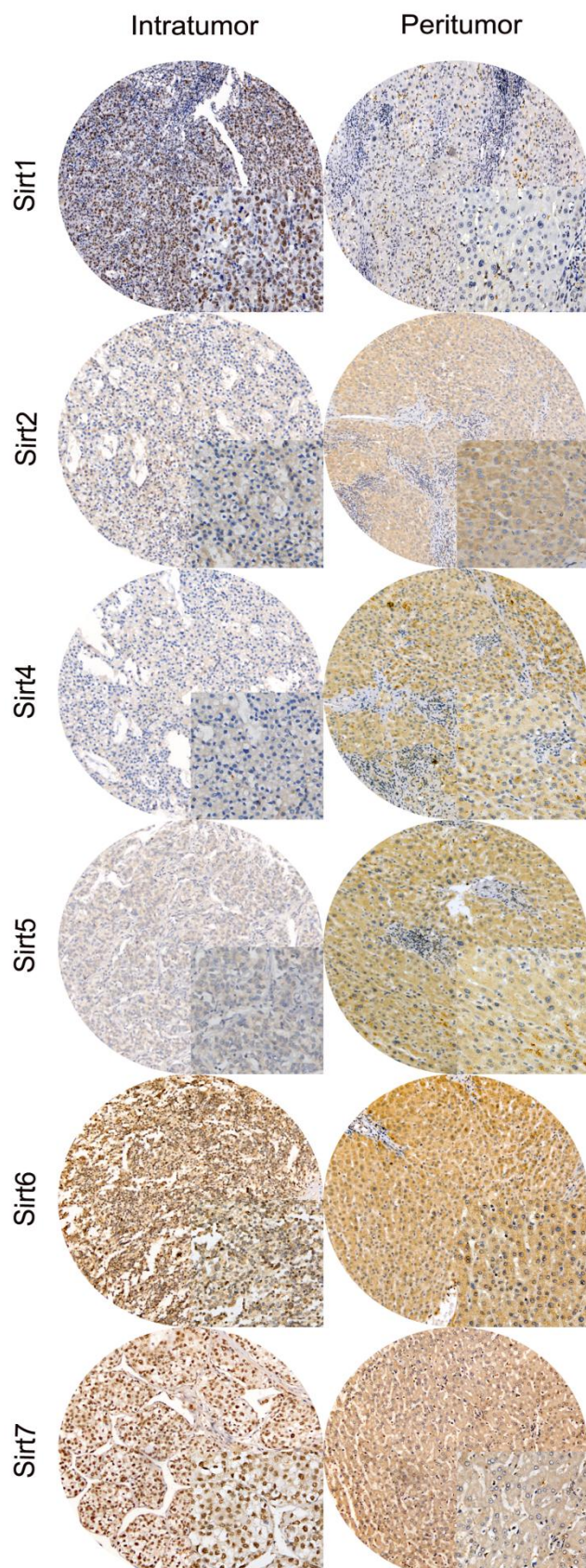

Supplement: Additional file 1: Figure S1 — Representative IHC staining of Sirt1, 2, 4, 5, 6, 7. The micrographs showed nearly negative cytoplasma staining of Sirt2, Sirt4, Sirt5 in tumor tissues, and Sirt1, Sirt6, Sirt7 of peritumoral liver tissues in HCC patients. (magnification100× & 400×). [file 1471-2407-14-297-S1.pdf]

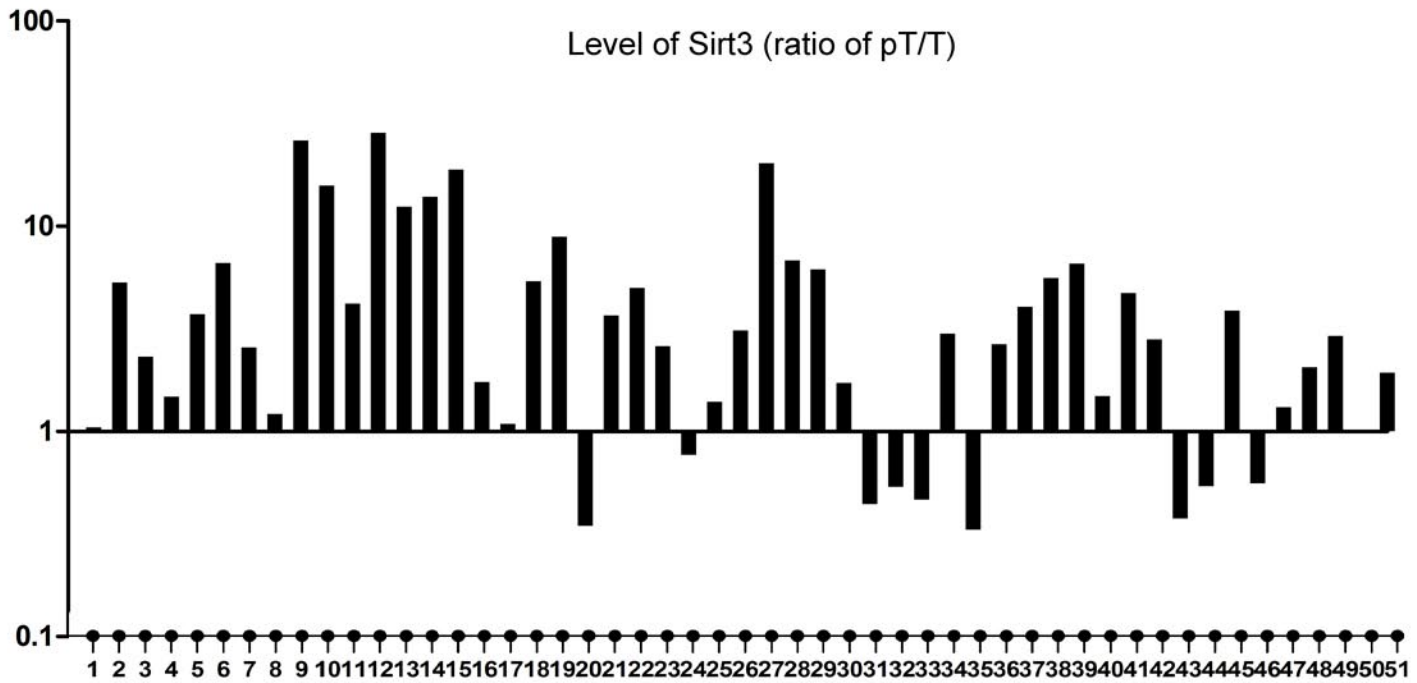

Supplement: Additional file 4 — The ratio of pT/T of Sirt3 by WB in 51 HCC patients. [file 1471-2407-14-297-S4.pdf]

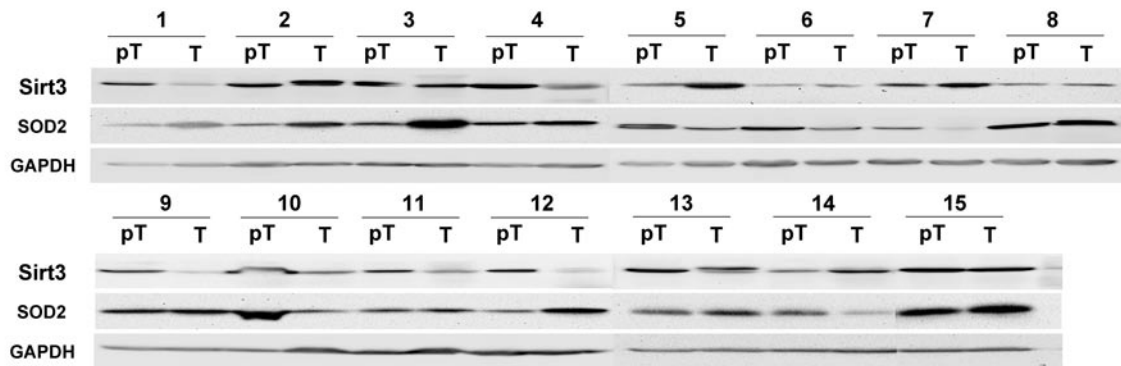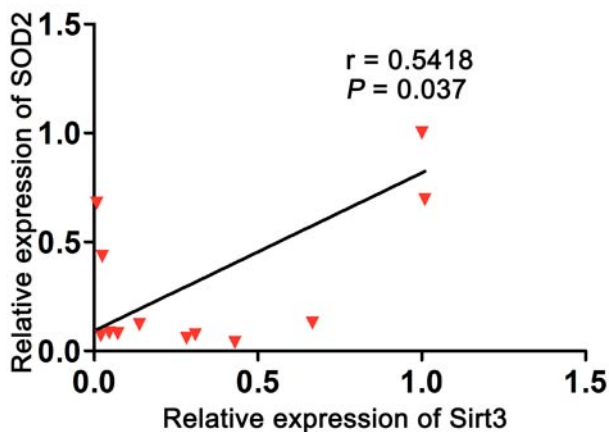

Supplement: Additional file 6 — Correlation between expression level of Sirt3 and SOD2 in HCC patients. 15 cases were studied. [file 1471-2407-14-297-S6.pdf]
