# Supplementary material for: Down-regulation of sirtuin 3 is associated with poor prognosis in hepatocellular carcinoma after resection
Source: BMC Cancer. 2014 Apr 28;14:297. doi: 10.1186/1471-2407-14-297 (PMC4021365; doi:10.1186/1471-2407-14-297)
Supplement: Additional file 2: Table S1 — X-tile for minimum P value. [file 1471-2407-14-297-S2.pdf]

Table S1. X-tile for minimum *P* value

***P* values for TTR (n=342)**

Score	iSirt1	pSirt1	iSirt2	pSirt2	iSirt3	pSirt3	iSirt4	pSirt4	iSirt5	pSirt5	iSirt6	pSirt6	iSirt7	pSirt7
0	0.752	1.000	0.121	0.439	0.069	0.294	0.752	0.655	0.655	0.752	0.294	0.094	0.439	1.000
1	0.752	1.000	0.527	0.371	0.157	0.317	0.371	0.752	0.403	0.752	0.584	0.138	0.371	1.000
2	1.000	0.752	0.074	0.138	0.011	0.527	0.051	0.014	0.036	0.752	1.000	0.114	0.527	0.147
3	1.000	0.129	0.584	0.403	0.129	0.254	0.254	0.371	0.034	0.192	1.000	0.016	0.206	0.107
4	0.273	1.000	0.480	0.192	1.000	0.016	0.403	0.019	0.584	0.129	0.655	1.000	0.051	0.051
5	0.094		0.403	0.439	0.403	1.000	1.000	0.094	0.403	0.221	1.000		0.273	0.752
6	0.752		1.000	1.000	1.000	1.000		0.254	1.000	0.584	1.000		0.317	0.480
7	1.000		1.000					1.000		1.000			0.317	1.000
8													1.000	

***P* values for OS (n=342)**

Score	iSirt1	pSirt1	iSirt2	pSirt2	iSirt3	pSirt3	iSirt4	pSirt4	iSirt5	pSirt5	iSirt6	pSirt6	iSirt7	pSirt7
0	0.655	0.752	0.371	0.480	0.001	0.011	0.317	0.034	0.180	0.294	0.254	0.527	1.000	1.000
1	0.584	0.343	0.480	0.752	0.000	0.023	0.030	0.114	0.012	0.138	0.655	0.317	0.584	0.752
2	0.221	0.752	0.009	0.114	0.001	0.584	0.004	0.008	0.001	0.206	1.000	0.020	0.527	0.343
3	0.254	0.061	0.074	0.078	0.006	0.192	0.180	0.254	0.001	0.083	0.655	0.017	0.157	0.480
4	0.254	1.000	0.655	0.024	0.655	0.024	0.480	0.074	0.439	0.157	0.584	1.000	0.114	0.036
5	0.168		0.655	0.138	0.480	0.371	1.000	0.107	0.480	1.000	0.752		0.273	0.004
6	0.584		0.752	1.000	1.000	1.000		0.480	1.000	0.439	1.000		0.254	0.046
7	1.000		1.000					1.000		1.000			0.237	0.083
8													1.000	1.000

Note: OS, overall survival; TTR, time to recurrence.
