# Supplementary material for: Down-regulation of sirtuin 3 is associated with poor prognosis in hepatocellular carcinoma after resection
Source: BMC Cancer. 2014 Apr 28;14:297. doi: 10.1186/1471-2407-14-297 (PMC4021365; doi:10.1186/1471-2407-14-297)
Supplement: Additional file 3: Table S2 — Univariate and multivariate analyses of prognostic factors. [file 1471-2407-14-297-S3.pdf]

Table S2. Univariate and multivariate analyses of prognostic factors

	TTR			OS		
	Univariate	Multivariate		Univariate	Multivariate	
	P value	H.R. (95% CI)	*P* value	*P* value	H.R. (95% CI)	*P* value
Age, year (≤52 vs >52)	0.173	NA	NA	0.787	NA	NA
Gender (female vs male)	0.616	NA	NA	0.654	NA	NA
HBV infection (no vs yes)	0.903	NA	NA	0.168	NA	NA
Liver cirrhosis (no vs yes)	0.803	NA	NA	0.225	NA	NA
ALT, U/L (≤75 vs >75)	0.177	NA	NA	0.225	NA	NA
γ-GT, U/L (≤54 vs >54)	<0.001		0.008	<0.001		0.046
AFP, ng/ml (≤20 vs >20)	0.004		0.023	0.014		0.098
Tumor size, cm (≤5 vs >5)	<0.001		<0.001	<0.001		<0.001
Tumor number (single vs multiple)	0.057	NA	NA	0.069	NA	NA
Tumor capsule (yes vs no)	0.096	NA	NA	0.167	NA	NA
Differentiation (well vs poor)	0.162	NA	NA	0.124	NA	NA
Tumor thrombi (no vs yes)	0.001		0.021	<0.001	NA	0.009
TNM stage (I vs II/III)	<0.001	NA	NA	<0.001	NA	NA
BCLC stage (AvsB/C)	0.001	NA	NA	<0.001	NA	NA
Mean Density Score (low vs high)						
iSirt1 (2)	0.928		NA	0.217		NA
pSirt1 (3)	0.129		NA	0.064		NA
iSirt2 (2)	0.074		NA	0.008		0.265
pSirt2 (4)	0.408		NA	0.076		NA
iSirt4 (2)	0.052		NA	0.004	0.683(0.470-0.991)	0.042
pSirt4 (4)	0.019	0.708 (0.500-1.001)	0.047	0.075		NA
iSirt5 (3)	0.035		0.310	0.001	0.561(0.362-0.867)	0.006
pSirt5 (4)	0.130		NA	0.157		NA
iSirt6 (0)	0.310		NA	0.253		NA
pSirt6 (3)	0.016		0.050	0.017		0.081
iSirt7 (6)	0.319		NA	0.257		NA
pSirt7 (4)	0.120		NA	0.043	0.515(0.251-1.057)	0.039

Note: Univariate analysis was calculated by the Kaplan–Meier method (the log-rank test). Multivariate analysis was done using the Cox multivariate proportional hazards regression model in a stepwise manner (forward, likelihood ratio). AFP, a-fetoprotein; 95% CI, 95% confidence interval; γ-GT, γ-glutamyl transferase; HR, hazard ratio; NA, not applicable; OS, overall survival; TTR, time to recurrence.
