# Supplementary material for: Down-regulation of sirtuin 3 is associated with poor prognosis in hepatocellular carcinoma after resection
Source: BMC Cancer. 2014 Apr 28;14:297. doi: 10.1186/1471-2407-14-297 (PMC4021365; doi:10.1186/1471-2407-14-297)
Supplement: Additional file 5 — Correlation between the ratio of iSirt3/pSirt3 and clinicopathologic characteristics in 51 patients. [file 1471-2407-14-297-S5.pdf]

Additional file 5. Correlation Between the ratio of iSirt3/pSirt3 and Clinicopathologic Characteristics in 51 patients

Characteristic		Total	iSirt3/pSirt3		*p* value
			<1	>1	
recurrence	no	36	30	6	0.776
	yes	15	12	3	
BCLC stage	A	35	29	6	0.889
	B+C	16	13	3	

Note: χ^2 tests for all the analysis.
